# Genome-wide association study and Mendelian randomization analysis provide insights for improving rice yield potential

**DOI:** 10.1038/s41598-021-86389-7

**Published:** 2021-03-25

**Authors:** Jing Su, Kai Xu, Zirong Li, Yuan Hu, Zhongli Hu, Xingfei Zheng, Shufeng Song, Zhonghai Tang, Lanzhi Li

**Affiliations:** 1 Hunan Engineering & Technology Research Center for Agricultural Big Data Analysis & Decision-Making, Hunan Agricultural University, Changsha, 410128 China; 2 State Key Laboratory of Hybrid Rice, Wuhan University, Wuhan, 430072 China; 3 Hubei Key Laboratory of Food Crop Germplasm and Genetic Improvement, Food Crop Institute, Hubei Academy of Agricultural Sciences, Wuhan, 430064 China; 4 State Key Laboratory of Hybrid Rice, Hunan Hybrid Rice Research Center, Changsha, 410125 China; 5 College of Food Science and Technology, Hunan Agricultural University, Changsha, 410128 China

**Keywords:** Agricultural genetics, Plant breeding, Plant genetics

## Abstract

Rice yield per plant has a complex genetic architecture, which is mainly determined by its three component traits: the number of grains per panicle (GPP), kilo-grain weight (KGW), and tillers per plant (TP). Exploring ideotype breeding based on selection for genetically less complex component traits is an alternative route for further improving rice production. To understand the genetic basis of the relationship between rice yield and component traits, we investigated the four traits of two rice hybrid populations (575 + 1495 F_1_) in different environments and conducted meta-analyses of genome-wide association study (meta-GWAS). In total, 3589 significant loci for three components traits were detected, while only 3 loci for yield were detected. It indicated that rice yield is mainly controlled by minor-effect loci and hardly to be identified. Selecting quantitative trait locus/gene affected component traits to further enhance yield is recommended. Mendelian randomization design is adopted to investigate the genetic effects of loci on yield through component traits and estimate the genetic relationship between rice yield and its component traits by these loci. The loci for GPP or TP mainly had a positive genetic effect on yield, but the loci for KGW with different direction effects (positive effect or negative effect). Additionally, TP (Beta = 1.865) has a greater effect on yield than KGW (Beta = 1.016) and GPP (Beta = 0.086). Five significant loci for component traits that had an indirect effect on yield were identified. Pyramiding superior alleles of the five loci revealed improved yield. A combination of direct and indirect effects may better contribute to the yield potential of rice. Our findings provided a rationale for using component traits as indirect indices to enhanced rice yield, which will be helpful for further understanding the genetic basis of yield and provide valuable information for improving rice yield potential.

## Introduction

Rice is a staple food crop for about half of the world. Improving rice productivity has been the main goal of rice breeding research since the growth of population and the loss of arable land. However, rice yield per plant has a complex genetic architecture, which is determined by various physiological processes changing temporally during the growing period. These processes often matched the yield component traits that are genetically less complex than yield^1^. Therefore, selecting the component traits of yield was proposed as a complementary route for further improving rice production, which also has been emphasized by national and international rice breeding programs^2^. Studying the genetic relationship between rice yield and component traits, and selecting the component traits to improve rice yield, will provide new clues for enhancing rice yield potential.

Rice yield per plant is a very complex agronomic trait mainly determined by its three component traits: the number of grains per panicle (GPP), kilo-grain weight (KGW) and tillers per plant (TP), which are typical quantitative traits that are affected by multiple genes and the environment, with low heritability^3^. With the development of high-throughput technology, a large number of genes/quantitative trait loci (QTLs) of the three component traits were identified using QTL mapping and genome-wide association study (GWAS) methods^4,5^. At the end of 2019, 209, 223, and 239 genes/QTLs for GPP (TO: 0000445), KGW (TO: 0000382), and TP (TO: 0000152) were identified respectively (http://www.gramene.org/), which densely distributed across the 12 chromosomes. Some of them have been applied in the rational design of super rice by marker-assisted selection (MAS) breeding, in which multiple defined genes with superior alleles pyramided to increase rice yield^6^. Liu et al. introduced the *DEP1* and *Gn1* genes introduced into the restorer line *93–11,* then the yield of the *DEP1 / Gn1-9311* line was significantly improved, due to resource allocation improved^7^. In 2020, Wang et al. compared the transgenic lines with *GNP1* or *NAL1* to the transgenic lines with both genes. They found the latter had a significantly higher yield, which indicated the two gene combinations may enhance the source-sink relationship^8^. In the above researches, only a small number of genes combined for super rice breeding, if more genes are selected for pyramiding, the trade-offs between different traits need to carefully consider^9^. Therefore, understanding the nature and strength of the relationship between yield per plant and its components will be helpful for efficient gene selection in MAS breeding^10^.

The relationship between rice yield per plant and its components was investigated by various researchers with different materials and methods, but they were inconsistent. In Huang et al.’s study, the superior alleles of grain number generally had a positive effect on yield, while the superior alleles of grain weight generally have a negative effect on yield^11^. Path analyses were performed by Oladosu et al. on rice yield and component traits revealed that three component traits possessed a positive effect with yield^12^. Xu et al. conducted a correlation analysis between yield and its components of 300 rice germplasms. Their result indicated that yield was significantly correlated with GPP or KGW, but non-significant correlations of yield were found with TP^13^. One possible explanation for the conflicting results is that the bias caused by the small sample size and lack of proper control for potential unmeasured confounders. For allowing the synthesis of results from different studies to estimate a common summary effect, the meta-analysis was recognized as the appropriate method to achieve adequate sample sizes and optimal power^14^. Meta-analysis of GWAS is powerful in dissecting complex human diseases. It is the statistical synthesis of information from multiple cohorts independent GWAS studies, which increases power and reduces false-positive findings^15^. Compared to humans, plants were planted in multiple years, environments, and locations. Meta-analysis is a useful way to narrowing down confidence intervals of QTL by compiling QTL information from multiple years and locations^16^. A recent meta-analysis of GWAS in tomato demonstrated the benefits obtained from meta-analysis in plants. Meta-analysis can assess the heterogeneity of studies, which can be caused by many factors, such as phenotypic structure, genetic structure, linkage disequilibrium, imputation accuracies or interaction between genotype and environment^17,18^.

Recently, the Mendelian randomization (MR) approach is a popular technique to assess the causal relationship between disease and environmental risk factors within a meta-analysis framework in epidemiology^19^. MR method was used to investigate the role of ATP citrate lyase inhibitors in cardiovascular disease^20^, in which the potential unmeasured confounders could be well protected from the observed association. In the MR approach, genetic variants were used as instrumental variables to avoid the possibility of confounding, because the genetic variants are randomly allocated at meiosis^21^. Thus, combine meta-analysis and MR for complex traits will help researchers to obtain a more reliable conclusion of their genetic relationship and further understand the genetic basis of rice yield.

GWAS has been proved to be a new strategy for explaining the genetic basis of complex traits, which has the advantage of improving the efficiency of detecting natural variations^22^. Most GWAS studies focused on dissecting the genetic basis of single yield traits^23,24^, but the study on clarifying the genetic basis of the relationship between the yield and component traits of rice is few. Here we carried out meta-analyses of GWAS results from two populations (575 + 1495 F_1_) in different environments and adopted an MR design to further estimated the genetic relationship between yield per plant (YD) and component traits of rice. We aimed to detect significant single-nucleotide polymorphisms (SNPs) associated with yield or component traits, to analyze the genetic bases contributing to the relationship between them, and to investigate possible utilization pattern for selecting the component traits of yield in breeding practice to further understand the genetic basis of yield and improve the rice production. The study will provide theoretical guidelines for enhancing rice yield potential.

## Results

### Meta-GWAS analyses

**Figure 1:**
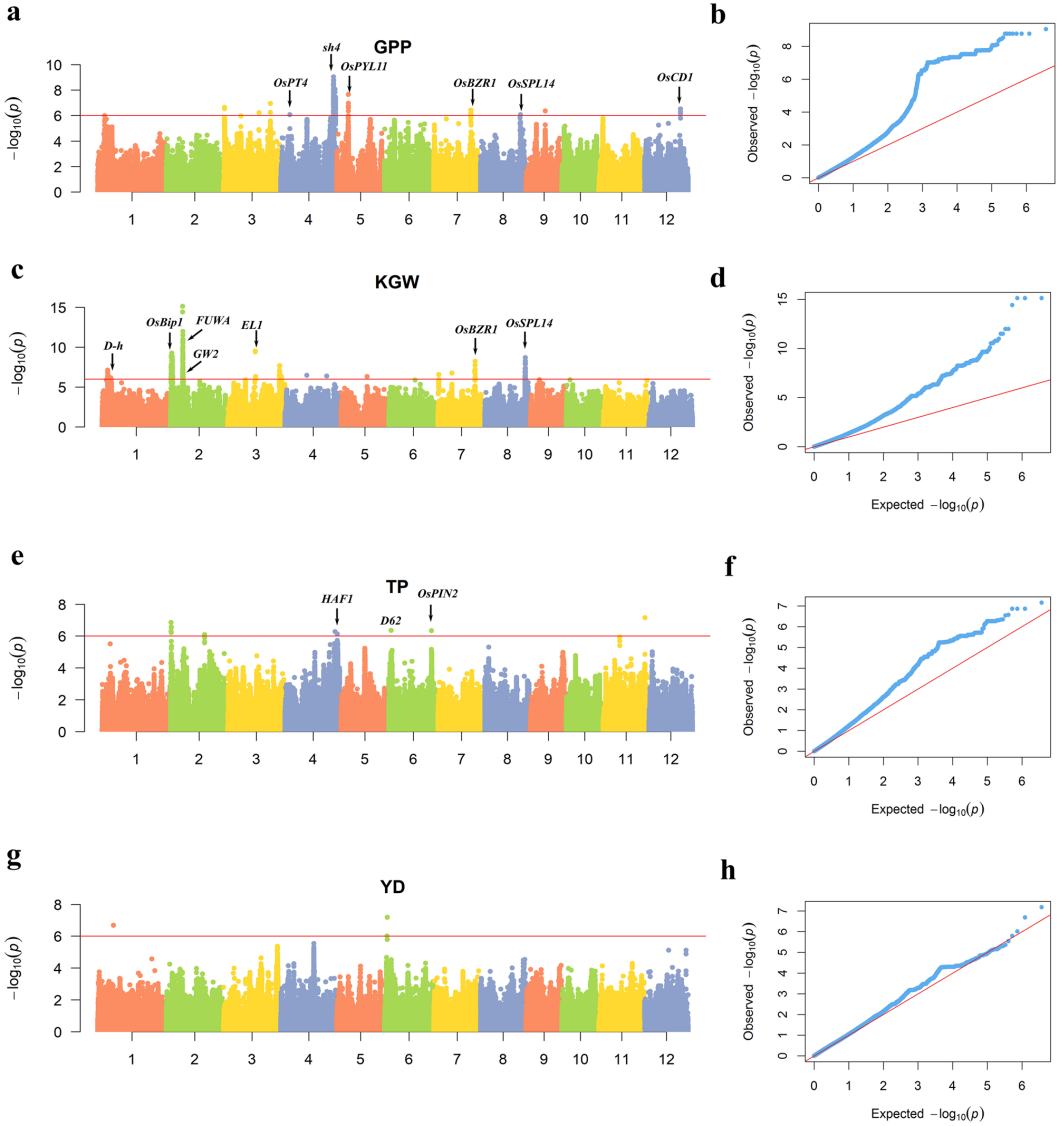
Meta-analyses result for GWAS. (**a**,**b**) Manhattan plots and quantile–quantile plots of GPP. (**c**,**d**) Manhattan plots and quantile–quantile plots of KGW. (**e**,**f**) Manhattan plots and quantile–quantile plots of TP. (**g**,**h**) Manhattan plots and quantile–quantile plots of YD. The genome-wide significant *P-*value threshold *P* < 10^–6^ is indicated by a horizontal line. The loci with well-characterized genes are indicated near the association peaks.

Meta-analyses of GWAS were performed based on four datasets’ (two locations for each population) GWAS results (Supplementary Figs. S1–S4). Manhattan plots and quantile–quantile plots of meta-GWAS are shown in Fig. 1. A total of 3592 significant loci were identified (Supplementary Table S1), including 2450, 1116, 23 and, 3 significant associated loci were separately detected for GPP, KGW, TP, and YD, which were distributed on all of the rice chromosomes except for chromosome 10. According to the information of RAP-DB (http://rapdb.dna.affrc.go.jp/), candidate genes were searched in a genomic region of 200 kb around the associated SNPs (Supplementary Table S2). We discovered 6, 7 and 3 cloned genes separately associated with GPP, KGW and TP. A total of three candidate genes associated with different traits, among which *OsBZR1* and *OsSPL14* have been reported previously^25,26^, and *Os02g0106966* was novelty discovered. This gene was annotated as a gene similar to *EMB1507* (embryo defective). Many embryo defective genes were identified in Arabidopsis^27^ (David W. Meinke, 2020), among which *EMB1507* caused the embryo lethal phenotype^28^. Both *OsBZR1* and *OsSPL14* were detected in KGW and GPP, *Os02g0106966* was detected in KGW and TP. In this study, only 3 significant loci for YD were detected, but 3589 significant loci for the component traits were detected. It may be because rice yield has a low heritability which is mainly affected by many minor-effect genes, the low heritability of rice yield is also showed in our previous study^29^. These results suggested that selecting the component traits of yield as a complementary route to improve rice production is recommended.

### The genetic relationship between GPP and YD

**Figure 2:**
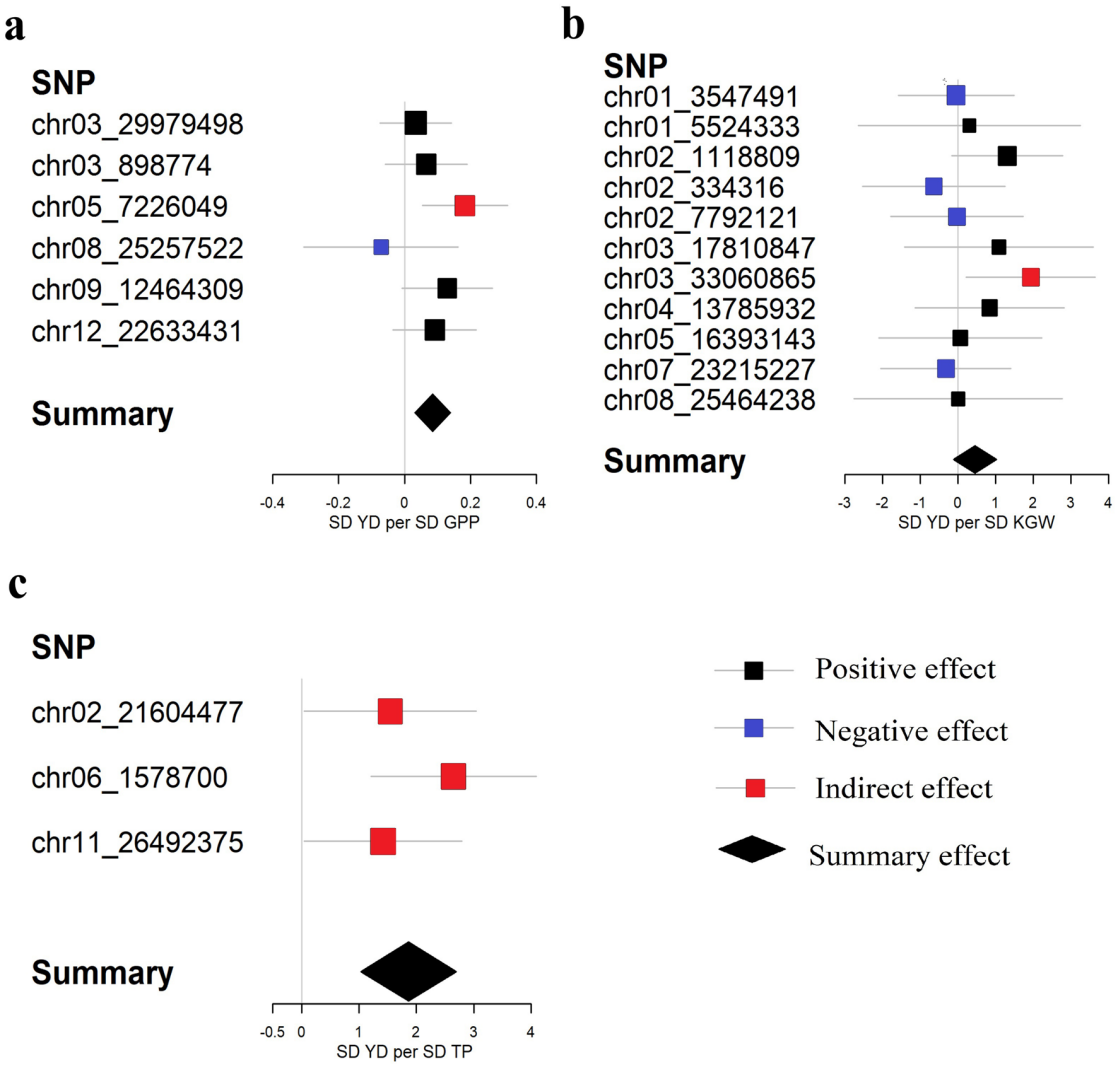
Genetic effect estimates of yield and its components. Estimates are derived from IVW method of MR analyses. (**a**) Effect estimates between GPP and YD. (**b**) Effect estimates between KGW and YD. (**c**) Effect estimates between TP and YD.

As required for MR analysis, a total of 2450 SNPs reached genome-wide significance for GPP (*P* < 1E−06) in meta-analyses of GWAS, among which 16 SNPs were not associated with KGW or TP (*P* > 0.05). We calculated the *r*^*2*^ of all pairs between the 16 SNPs, and then discarded SNPs in LD (*r*^*2*^ > 0.01) based on larger *P-*value. The remaining six SNPs were selected as instrumental variables to estimate the genetic relationship between GPP and YD (Table 1). For MR analysis, these loci mainly had a positive genetic effect on yield through GPP and a positive genetic relationship between GPP and YD were observed with the inverse-variance weighting (IVW) method (Table 4, Fig. 2a). One standard deviation (SD) genetic higher GPP was associated with a 0.086 SD higher YD (Beta = 0.086, 95% CI: 0.030 ~ 0.141, *P* = 0.003). In sensitivity analyses, Cochran's Q-test illustrated no obvious heterogeneity (*I*^*2*^ = 5%, *P* = 0.38). The weighted median method also showed GPP had a positive effect on YD (Beta = 0.081, 95% CI: 0.009 ~ 0.152, *P* = 0.028). MR-Egger regression indicated no evidence of directional pleiotropy for the associations of GPP with YD (intercept = 1.387, *P* = 0.061). It is worth noting that some cloned genes were detected in the meta-GWAS on GPP, the phenotype of transgenic plants with these genes had a similar phenomenon. For example, the *OsSPL14* mutant produced more grain number per panicle, enhanced rice yield^26^. Compared with the control non-transgenic plants, the over-expression of *OsBZR1* plants showed the 1000-grain weight was increased by about 3.4% and the spikelet number per panicle was increased 21.9%, that resulting in enhanced yield^25^. The *cd1* mutant exhibited a variety of phenotypic traits, such as a reduction in grain number and panicle length, the biomass was lower than that of the wild type^30^.

**Table 1:** Information about instrumental variables. All the SNP markers are named after the chromosome_position.

SNP	Chromosome	Position	GPP		YD	
			Beta	*P*-value	Beta	*P*-value
chr03_29979498	3	29,979,498	− 18.724	1.12E−07	− 0.633	0.543
chr03_898774	3	898,774	18.953	2.92E−07	1.254	0.299
chr05_7226049	5	7,226,049	− 7.242	2.25E−08	− 1.329	0.005
chr08_25257522	8	25,257,522	− 16.559	8.58E−07	1.178	0.553
chr09_12464309	9	12,464,309	− 7.538	4.49E−07	− 0.974	0.066
chr12_22633431	12	22,633,431	15.738	3.01E−07	1.439	0.158

### The genetic relationship between KGW and YD

**Figure 3:**
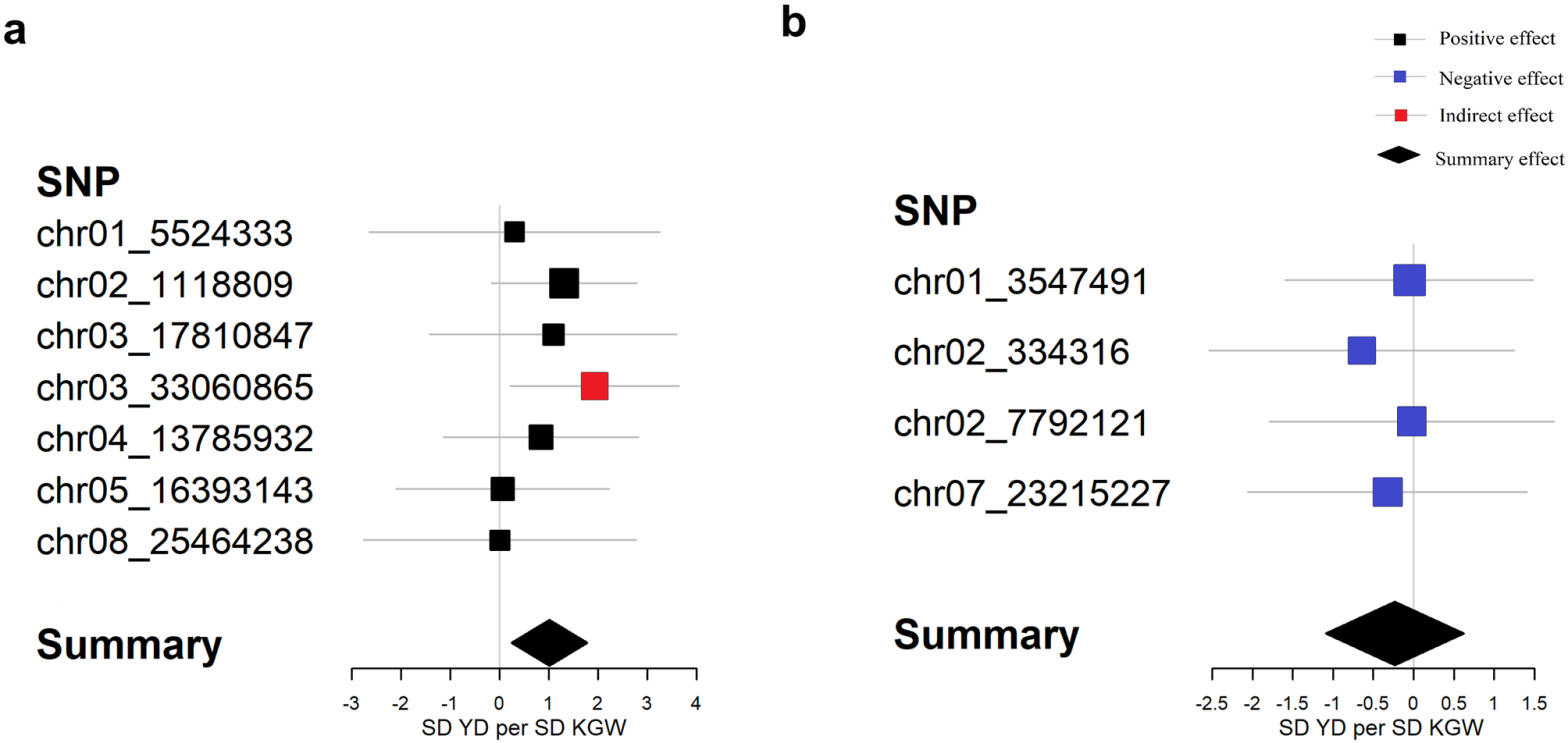
Genetic effect estimates of KGW and YD. Estimates are derived from IVW method of MR analyses. (**a**) Positive effect estimates between KGW and YD. (**b**) Negative effect estimates between KGW and YD.

As required for MR analysis, a total of 1116 SNPs reached genome-wide significance for KGW (*P* < 1E−06) in meta-analyses of GWAS, among which 395 SNPs were not associated with GPP or TP (*P *> 0.05), we calculated the *r*^*2*^ of all pairs between the 395 SNPs, and discarded SNPs in LD (*r*^*2*^ > 0.01) based on larger *P-*value. The remaining eleven SNPs were selected as instrumental variables to estimate the genetic relationship between KGW and YD (Table 3). For MR analysis, we observed that a part of SNP for KGW had a positive effect on YD, a part of SNP for KGW had a negative effect on YD (Table 2, Fig. 2b). To further understand the genetic relationship between KGW and YD, the SNPs with different directions of genetic effects were studied separately in our study. These loci with positive effect showed that KGW had a positive effect on yield, while these loci with negative effect showed that KGW had no significant negative effect on yield (Fig. 3). In sensitivity analyses, Cochran's Q-test illustrated no obvious heterogeneity (*I*^*2*^ = 0%). The weighted median method also confirmed the results of the IVW method. MR-Egger regression indicated no evidence of pleiotropy for the associations of KGW with YD (Table 4). The cloned gene *GW2* was detected in the meta-GWAS on KGW has been reported to have the potential to enhance rice yield^31^.

**Table 2:** Information about instrumental variables.

SNP	Chromosome	Position	KGW		YD	
			Beta	*P*-value	Beta	*P*-value
chr01_3547491	1	3,547,491	0.706	7.14E−08	− 0.036	0.948
chr01_5524333	1	5,524,333	1.018	6.56E−07	0.310	0.840
chr02_1118809	2	1,118,809	0.786	1.56E−09	1.030	0.082
chr02_334316	2	334,316	− 1.474	2.38E−07	0.948	0.505
chr02_7792121	2	7,792,121	− 1.256	9.21E−08	0.029	0.980
chr03_17810847	3	17,810,847	0.651	4.74E−07	0.711	0.394
chr03_33060865	3	33,060,865	− 0.505	2.03E−08	− 0.978	0.027
chr04_13785932	4	13,785,932	0.739	3.16E−07	0.623	0.406
chr05_16393143	5	16,393,143	0.466	4.71E−07	0.029	0.955
chr07_23215227	7	23,215,227	0.952	1.09E−07	− 0.308	0.715
chr08_25464238	8	25,464,238	− 0.875	2.01E−09	− 0.004	0.997

### The genetic relationship between TP and YD

As required for MR analysis, a total of 23 SNPs reached genome-wide significance for TP (*P* < 1E−06) in meta-analyses of GWAS, among which four SNPs were not associated with KGW or GPP (*P* > 0.05), we calculated the *r*^*2*^ of all pairs between the four SNPs, and discarded SNPs in LD (*r*^*2*^ > 0.01) based on larger *P-*value. The remaining three SNPs were selected as instrumental variables to estimate the genetic relationship between TP and YD (Table 3). For MR analysis, these loci had a positive genetic effect on yield through TP and a positive genetic relationship between TP and YD were observed with the IVW method (Table 4, Fig. 2c), 1 SD genetic higher TP was associated with a 1.865 SD higher YD (Beta = 1.865, 95% CI: 1.035 ~ 2.694, *P* < 0.0001). Compared with KGW (Beta = 1.016) and GPP (Beta = 0.086), TP (Beta = 1.865) has a greater effect on yield. In sensitivity analyses, Cochran's Q-test illustrated no obvious heterogeneity (*I*^*2*^ = 0%, *P* = 0.43). The weighted median method also showed TP had a positive effect on YD (Beta = 1.54, 95% CI: 0.353 ~ 2.727, *P* = 0.011). MR-Egger regression indicated no evidence of directional pleiotropy for the associations of TP with YD (intercept = 0.046, *P* = 0.968). The cloned gene *OsPIN2* was detected in the meta-GWAS on TP. Chen et al. found that the *OsPIN2* transgenic plants had a more effective tiller number, lower 1000-grain weight, and higher yield^32^.

**Table 3:** Information about instrumental variables.

SNP	Chromosome	Position	TP		YD	
			Beta	*P*-value	Beta	*P*-value
chr02_21604477	2	21,604,477	− 1.279	8.25E−07	− 1.977	0.043
chr06_1578700	6	1,578,700	− 0.639	4.52E−07	− 1.691	3.21E−04
chr11_26492375	11	26,492,375	− 0.507	7.04E−08	− 0.720	0.043

**Table 4:** MR results of the relationship between yield and its component traits.

Trait	Methods	Beta	95% CI	*P*
GPP	IVW	0.086	0.030 ~ 0.141	0.003
	Weighted median	0.081	0.009 ~ 0.152	0.028
	MR-Egger	− 0.029	− 0.160 ~ 0.103	0.668
	MR-Egger(intercept)	1.387	− 0.063 ~ 2.836	0.061
KGW (positive)	IVW	1.016	0.242 ~ 1.791	0.010
	Weighted median	1.123	0.122 ~ 2.124	0.028
	MR-Egger	0.480	− 2.743 ~ 3.704	0.770
	MR-Egger(intercept)	0.349	− 1.690 ~ 2.388	0.737
KGW (negative)	IVW	− 0.233	− 1.092 ~ 0.626	0.595
	Weighted median	− 0.156	− 1.150 ~ 0.839	0.759
	MR-Egger	− 0.710	− 3.853 ~ 2.434	0.658
	MR-Egger(intercept)	0.464	− 2.480 ~ 3.407	0.757
TP	IVW	1.865	1.035 ~ 2.694	< 0.0001
	Weighted median	1.540	0.353 ~ 2.727	0.011
	MR-Egger	1.797	− 1.633 ~ 5.228	0.304
	MR-Egger(intercept)	0.046	− 2.165 ~ 2.256	0.968

*CI* confidence intervals, *P* statistically significant associations with a *P* < 0.05

### Loci for component traits had an indirect effect on yield

We identified five significant loci that had an indirect effect on yield by MR analyses (Fig. 2, Supplementary Table S3). Among them, the SNP chr05_7226049 (Fig. 2a) for GPP had an indirect effect on yield and was located nearby the cloned gene *OsPYL11.* Kim et al. reported that compared with the control plants, the transgenic plants overexpressing *OsPYL11* showed no significant difference in tiller number, but the yield was severely reduced^33^. Our study indicated the yield severely reduced may be caused by the number of grains decreased. The SNP chr03_33060865 (Fig. 2b) for KGW is in the vicinity of the cloned gene *EL1*, which is a key regulator of the gibberellin response, Kwon et al. discovered the plants that loss of *EL1* showed the 500-grain weight and yield significantly reduced^34^. The SNP chr06_1578700 (Fig. 2c) for TP closed to the *D62* (a gene regulating tillers). Li et al. found that the tiller number of *D62* mutant rice was less than that of the wild type^35^. The SNPs chr02_21604477 and chr11_26492375 for TP also had indirect effects on yield (Fig. 2c, Supplementary Table S3), which were first detected in our research. The SNP chr02_21604477 is closest to *Os02g0567900* (2101 bp from it). This gene was annotated as a gene similar to H0818E04.14 protein and involved in nucleic acid binding (GO:0003676) and nucleotide binding (GO:0000166). The SNP chr11_26492375 is closest to *Os11g0660000* (12 bp from it). This gene was annotated as sodium/calcium exchanger membrane region domain containing protein, which regulated magnesium/proton exchanger (K03452) and also related to transmembrane transport (GO:0055085) and is integral to the membrane (GO:0016021). These findings provided new information for further improve rice yield potential.

### Pyramiding superior alleles of significant loci

**Figure 4:**
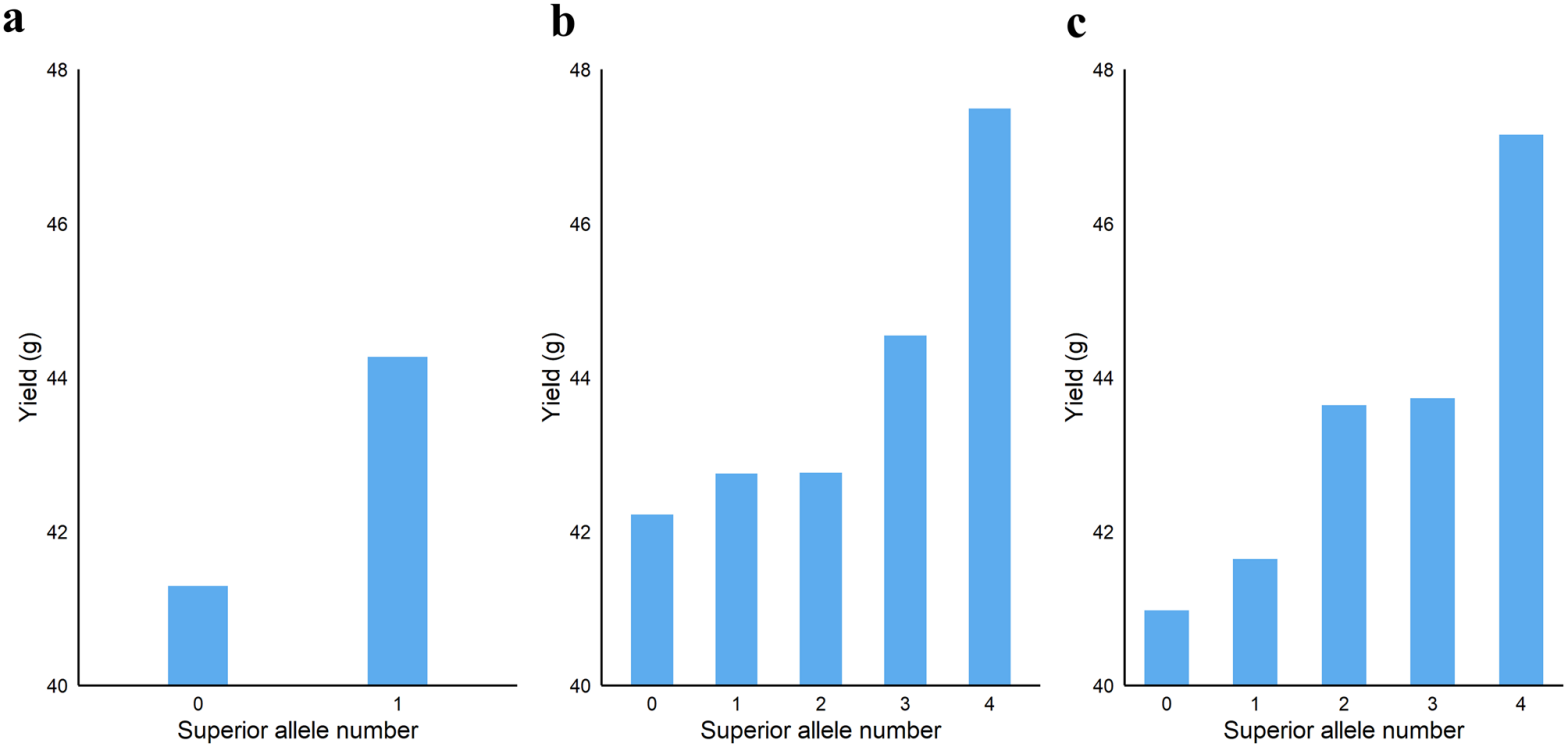
The average yield performance of F_1_ lines with different superior allele number of significant loci. (**a**) Direct loci, (**b**) Indirect loci, (**c**) Direct plus indirect loci.

The average yield performance of F_1_ lines with different superior allele numbers of significant loci with direct effect, indirect effect, and direct plus indirect effect was shown in Fig. 4. Three loci that had a direct effect on yield were detected in the meta-GWAS on YD (Supplementary Table S3), among them, the average yield of the lines without superior alleles was 41.29 g, and the average yield of the lines with one superior allele was 44.26 g (Fig. 4a, Supplementary Table S4). The superior alleles of five loci that had an indirect effect on yield were also pyramided in the study. The results showed that the average yield of F_1_ lines with 0 to 4 superior alleles was: 42.22 g, 42.75 g, 42.76 g, 44.54 g, 47.49 g, respectively. In general, the yield of F_1_ hybrids rises with increases of the superior alleles (Fig. 4b, Supplementary Table S4). A similar phenomenon was also found in pyramiding the direct plus indirect loci (Fig. 4c, Supplementary Table S4). Other research reported that the phenotype performance improved by pyramiding the superior alleles of loci associated with agronomic traits^11^, our results suggested the yield enhanced also by pyramiding the superior alleles of loci that had an indirect effect on yield. Hybrid lines pyramiding all the superior alleles of direct (3 loci) and indirect loci (5 loci) not be observed in this study. Our results indicated rice production improved with the increase of the superior alleles, it is possible that a combination of direct and indirect effects will better contribute to the yield potential of rice.

## Discussion

In this study, a total of 3592 significant SNPs were detected in meta-GWAS on yield or its component traits, which provide more information for rice agronomic traits breeding. It is worth noting that only 3 loci were detected in meta-GWAS on yield, this may be the results from that rice yield has a low heritability and minor-effect loci hardly to be detected. For a low-heritability trait (such as yield), highly correlated auxiliary traits (such as GPP, etc.) will help improve the selection of traits with low heritability since they reflecting a shared biological basis^36^.

MR model was carried out to investigate the genetic effects of loci on yield through component traits. The loci for GPP or TP mainly had a positive genetic effect on yield, which was consistent with previous studies^26,32^. The loci for KGW had a different direction of effects (positive effect or negative effect) on yield. Huang et al. conducted GWAS on 1495 hybrid rice lines and found the superior alleles of grain weight generally had a negative effect on yield^11^, but some genes that regulate KGW have been reported to have the potential to enhance rice yield, such as *GW7*^37^ and *GS5*^38^. To some extent, our results confirmed both of their findings, which indicated our study had greater power and more comprehensive by synthesizes different data. Then these loci were used to explore the relationship between rice yield and its component traits through these loci by MR method. The results of MR provided some evidence that selecting the component traits of yield to improve rice production, which is consistent with the improvement of rice production achieved by genetic manipulation of the component traits in previous studies^37,39^. The MR analyses provided a rationale for using component traits as indirect indices to enhanced rice yield.

Five loci were identified with an indirect effect on yield by MR analysis, providing new information for enhancing the yield potential of rice. A previous study indicated that pyramiding the superior alleles of significant associated loci increased yield^11^. Our results suggested the improvement of yield also by pyramiding the superior alleles of loci with an indirect effect on yield. In this study, due to the limitation of sample size, hybrid lines pyramiding all the superior alleles of direct (3 loci) and indirect loci (5 loci) not be observed. However, it is interesting to note that the average yield performance of hybrid lines with 1 to 3 superior alleles of indirect loci improved with an increase of the superior alleles when adding superior alleles of the direct locus to the pyramid, the average yield performance of hybrid lines was increased. Similar to the performance of the superior alleles of four indirect loci, the yield of the superior alleles of five direct plus indirect loci also be improved (Fig. 4). Our results indicated rice production improved with the increase of the superior alleles, it is possible that a combination of direct and indirect effects will better contribute to the yield potential of rice. In addition, Huang et al. revealed numerous superior alleles that contribute to heterosis by the genomic analysis of hybrid rice varieties. They concluded that the accumulation of numerous rare superior alleles with positive dominance is an important contributor to the heterotic phenomena^11^. In our study, we also found some superior alleles for improvement of rice yield, which may contribute to the parent selection of hybrids. For example, the superior allele of the SNP chr01_9982003 is AA in the F_1_ population, which suggested that to obtain higher yields hybrid, parents’ allele genotype with AA at this locus is preferred. The superior allele of SNP chr06_1780896 is TC in the F_1_ population. It indicated that the genotype at the locus chr06_1780896 with the TT parent and the CC parent hybridization would produce a relatively high yield progeny. It would be possible to generate higher-yielding lines by combinations of parents selected according to these superior alleles.

The strengths of the study are: (i) a meta-analysis of GWAS data from multiple population and environments to estimate a summary effect provided greater statistical power^14^; (ii) The MR approach could less prone to confounders since the genetic variants used as instrumental variables^21^; (iii) Using MR method to analyze the genetic relationship between quantitative traits in this study, which weighted the effects of multiple independent SNPs into a summary effect, for quantitative traits, most of them are affected by multiple genes or the interaction of genes, while the individual SNP only explain a small fraction of the variation in the quantitative traits. Since the MR analysis may be biased by the possibility of invalid instrumental variables, it is difficult to completely exclude type I error and the potential influence of pleiotropy since the instrumental variables derived from the meta-analysis of GWAS in the study. Thus, we conducted a weighted median method and the MR-Egger method to do sensitivity analysis. Compared to the IVW method^40^, the weighted median method showed better finite-sample Type I error rates. The estimator was consistent even if up to 50% of the information comes from invalid instrumental variables^41^. The results of the MR-Egger and heterogeneity test indicated the genetic variants had no pleiotropic effects on yield to some extent^42^. These results strengthened our confidence in the validity of assumptions.

In conclusion, we analyzed the genetic basis of the relationship between yield and its component traits by GWAS and MR methods, providing genetic insights for further improving rice yield potential. Our results suggested the improvement of rice production by pyramiding the superior alleles of genes regulating component traits, and a combination of direct and indirect effects may better contribute to the yield potential of rice in breeding practice. These findings will provide theoretical guidelines for the rational design of rice by MAS breeding.

## Methods

### Materials and phenotyping

Two populations of rice hybrid varieties were used in our study. One of the populations consists of 575 F_1_ hybrid rice lines, which produced by 115 varieties (restorer lines of 29 three-line wild-deficient hybrid rice and 86 accessions of micro-core germplasm) as male parents were crossed with 5 sterile lines (4 two-line sterile lines and 1 three-line sterile line) as female parents. The 575 hybrid lines were grown both in Huazhong Agricultural University and Wuhan University in 2012. The other population from the national center for gene research of Chinese academy of sciences, which including 1,170 lines were bred from the three-line system and 325 lines were generated from the two-line system. The 1495 hybrid lines were grown in Hangzhou and Sanya respectively^11^. Genotypic and phenotypic data of the 1495 lines were downloaded for subsequent analysis in this study. A total of four agronomic traits including GPP, KGW, TP, and YD were recorded in both populations. The phenotyping standards for these agronomic traits are the same in both populations, which were measured for at least three samples of each accession, and the average measurement was taken as the phenotypic value for GWAS analysis.

### Resequencing and genotyping

The population of 575 hybrid rice lines was sequenced on the Illumina HiSeq2500 platform at 11 × genome coverage on average. By quality control, we obtained 1,894,012 high quality SNPs with minor allele frequency (MAF) > 5% and missing rate < 20% across the 575 accessions. The high diversity SNP maps of 1495 hybrid rice varieties are publicly available (http://www.ncgr.ac.cn/RiceHap4). The genomes of 1495 hybrid lines were sequenced on the Illumina HiSeq2000 at twofold genome coverage, and 1,531,463 SNPs passing quality control (MAF > 1%).

### Genotype imputation and GWAS analysis

3000 rice genomes project (https://snp-seek.irri.org/download.zul) as the reference panel was used to perform SNP imputation in the genotype data of 575 and 1495 hybrid rice lines by using beagle software (version 5.0)^43^, and all imputed SNPs with MAF < 1% were filtered. Among the 3000 rice genomes project^44^, the 4.8mio filtered SNP dataset is used as the reference panel in the study, with an average sequencing depth of 14 × and all SNP passed the quality control (MAF > 1%, missing rate = 0%). Then conducting separate GWAS for two populations in two different environments using mixed-linear-model association (MLMA) in GCTA software^45^ and collecting the summary statistics to run a meta-GWAS.

### Meta-GWAS analyses

Meta-GWAS is a meta-analysis of summary data (beta, standard error and *p*-values of each SNP) from each GWAS results. Imputation increased the genome-wide SNP densities, a total of 1,838,525 common SNPs from four GWAS datasets were used for meta-analysis. We used the fixed-effect model in METAL as the primary approach to conduct the meta-analyses^46^. The fixed-effect model adopts the inverse variance weighting method, which weighted each study according to the inverse of its squared standard error. Then the Cochran's Q-test was performed to heterogeneity test^47^. For those SNPs where heterogeneity occurs (*I*^*2*^ >  = 50%), the random effect model in METASOFT was adopted^48^ (Han et al. 2011). The genome-wide significant *P*-value for meta-GWAS was set as *P* < 1E−06 (− log_10_*P* = 6). According to the information of RAP-DB (http://rapdb.dna.affrc.go.jp/), candidate genes were searched in a genomic region of 200 KB around the associated SNPs. If there was a cloned gene reported to be related to yield-traits in a 200 kb genomic region, the cloned gene would be selected as the candidate gene; if not, the gene closest to the significant SNP would be selected as the candidate gene.

### MR analysis

For the genetic effect of rice yield and each component trait to be consistently estimated, the genetic variants were selected according to the three assumptions in MR analysis^38^, (i) the genetic variants were obtained from the results of meta-GWAS that associated with the single component trait at a genome-wide significant level (*P* < 1E−06); (ii) the genetic variants are not associated with any confounders; (iii) the genetic variants only affect yield through the single component trait, not through other component traits (*P* > 0.05). Since the selected SNPs in linkage disequilibrium (LD) may result in confounding^21^, we calculated the *r*^*2*^ (LD) of all pairs between all selected SNPs using plink (version 1.90)^49^ and discarded SNPs in LD (*r*^*2*^ > 0.01) based on larger *P-*value.

The IVW method was conducted for MR analysis to assess the effect of component traits on yield, which by summarizing the effects of multiple independent SNPs^38^. In sensitivity analyses, the weighted median method^39^ and MR-Egger method^40^ are used for MR analysis, which is more robust due to pleiotropic or invalid instruments involved.

### Analysis of superior alleles of significant associate loci

Calculated the average phenotypic measurement corresponding to genotypes of each significant SNP, and the least significant difference method was used for multiple comparisons. Following Huang et al.’s method^11^, the genotype of SNP with the highest-level yield or component trait was set to be the superior allele (for example, the allele corresponding to the largest number of grains per panicle was set to be the superior allele). Calculated the number of superior alleles in each hybrid rice line and recorded their corresponding average yield measurements. Omitted the number of superior alleles with less than 3 hybrid lines.

## Supplementary Information

Supplementary Information 1.

Supplementary Information 2.

## Data Availability

The datasets supporting the conclusions of this article are provided within the article and its electronic supplementary material, the datasets and the code used to execute the GWAS are available from the corresponding author on reasonable request.

## Abbreviations

GPP: The number of grains per panicle
KGW: Kilo-grain weight
TP: Tillers per plant
GWAS: Genome-wide association study
QTL: Quantitative trait locus
MAS: Marker-assisted selection
MR: Mendelian randomization
SNP: Single-nucleotide polymorphism
YD: Yield
MAF: Minor allele frequency
IVW: Inverse-variance weighting
LD: Linkage disequilibrium
CI: Confidence intervals
SD: Standard deviation
