# Supplementary material for: Genome-wide association study and Mendelian randomization analysis provide insights for improving rice yield potential
Source: Sci Rep. 2021 Mar 25;11:6894. doi: 10.1038/s41598-021-86389-7 (PMC7994632; doi:10.1038/s41598-021-86389-7)
Supplement: Supplementary file 1 — Supplementary Information 1. [file 41598_2021_86389_MOESM1_ESM.pdf]

Genome-wide association study and Mendelian randomization analysis provide insights for improving rice yield potential

Jing Su[1], Kai Xu[1], Zirong Li[1], Yuan Hu[1], Zhongli Hu[2], Xingfei Zheng[3], Shufeng Song[4], Zhonghai Tang[5], Lanzhi Li [1*]

[1]Hunan Engineering & Technology Research Center for Agricultural Big Data Analysis & Decision-making, Hunan Agricultural University, Changsha, 410128, China

[2]State Key Laboratory of Hybrid Rice, Wuhan University, Wuhan, 430072, China

[3]Hubei Key Laboratory of Food Crop Germplasm and Genetic Improvement, Food Crop Institute, Hubei Academy of Agricultural Sciences, Wuhan, 430064, China

[4]State Key Laboratory of Hybrid Rice, Hunan Hybrid Rice Research Center, Changsha 410125, China

[5]College of Food Science and Technology, Hunan Agricultural University, Changsha 410128, China

corresponding author

Lanzhi Li

Email: lancy0829@163.com

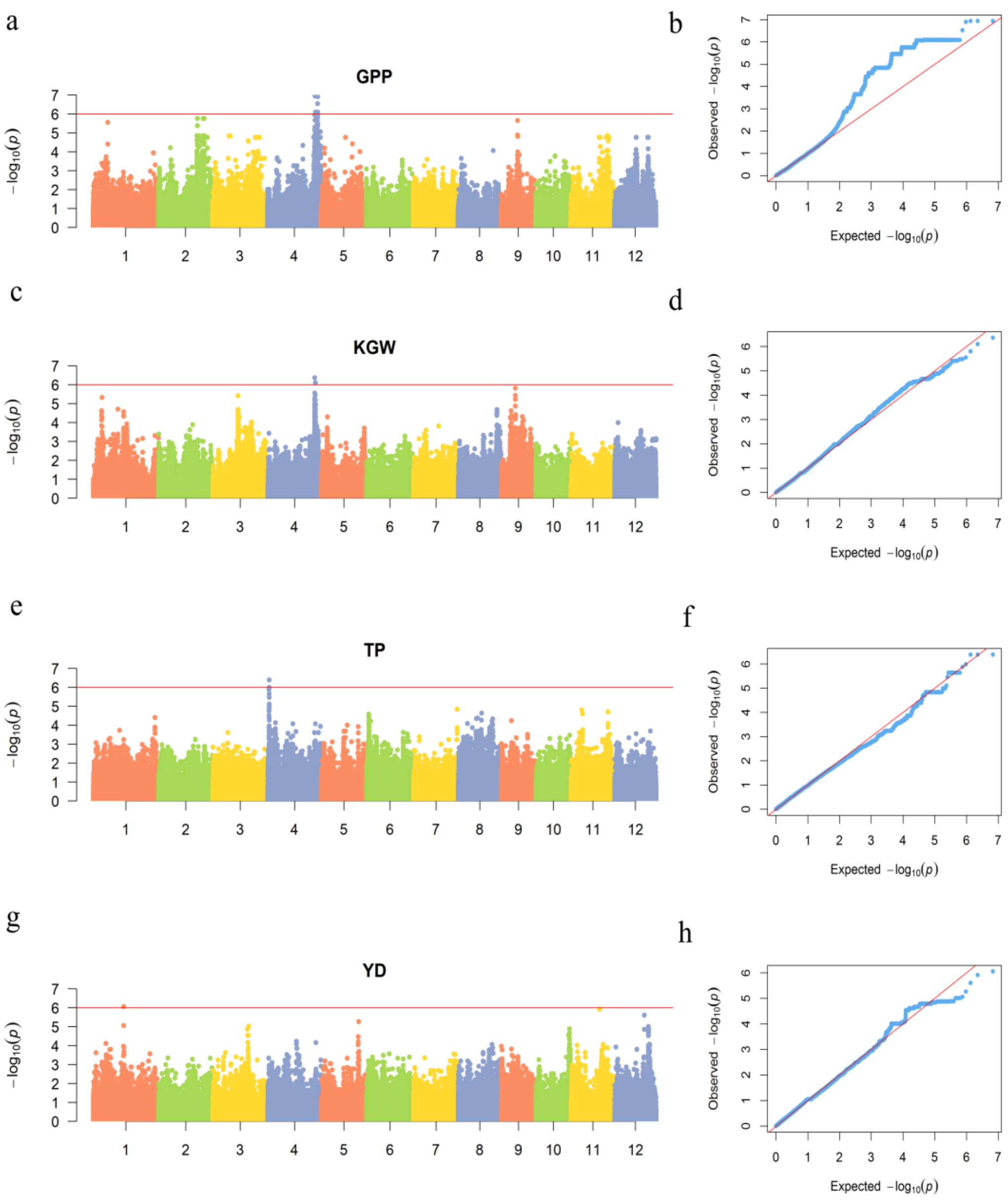

Figure S1. GWAS results of GPP, KGW, TP and YD in Huazhong Agricultural University. (a, b) Manhattan plots and quantile-quantile plots of GPP. (c, d) Manhattan plots and quantile-quantile plots of KGW. (e, f) Manhattan plots and quantile-quantile plots of TP. (g, h) Manhattan plots and quantile-quantile plots of YD. The genome-wide significant *P* value threshold $P<10^{-6}$ is indicated by a horizontal line.

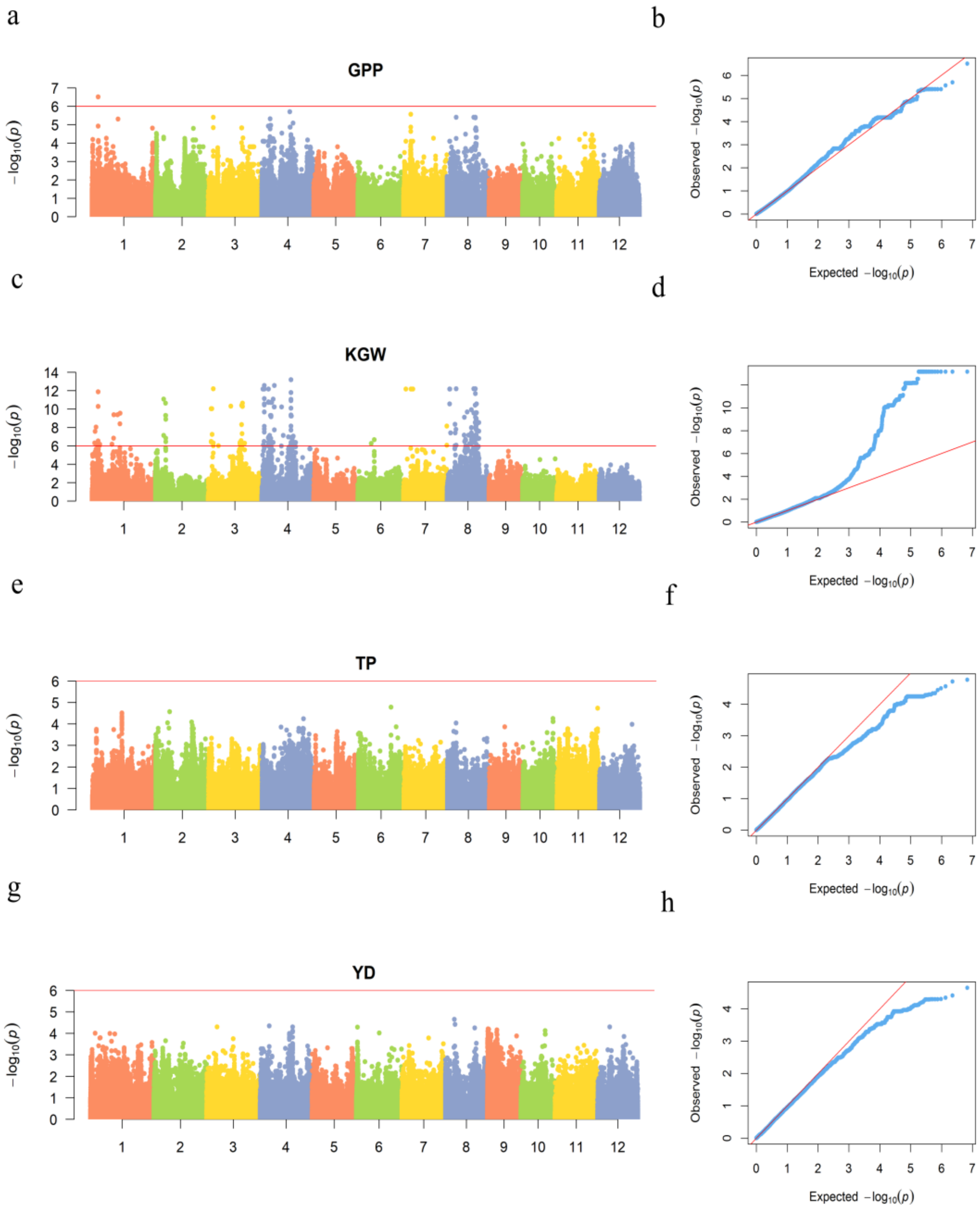

Figure S2. GWAS results of GPP, KGW, TP and YD in Wuhan University. (a, b) Manhattan plots and quantile-quantile plots of GPP. (c, d) Manhattan plots and quantile-quantile plots of KGW. (e, f) Manhattan plots and quantile-quantile plots of TP. (g, h) Manhattan plots and quantile-quantile plots of YD. The genome-wide significant *P* value threshold $P<10^{-6}$ is indicated by a horizontal line.

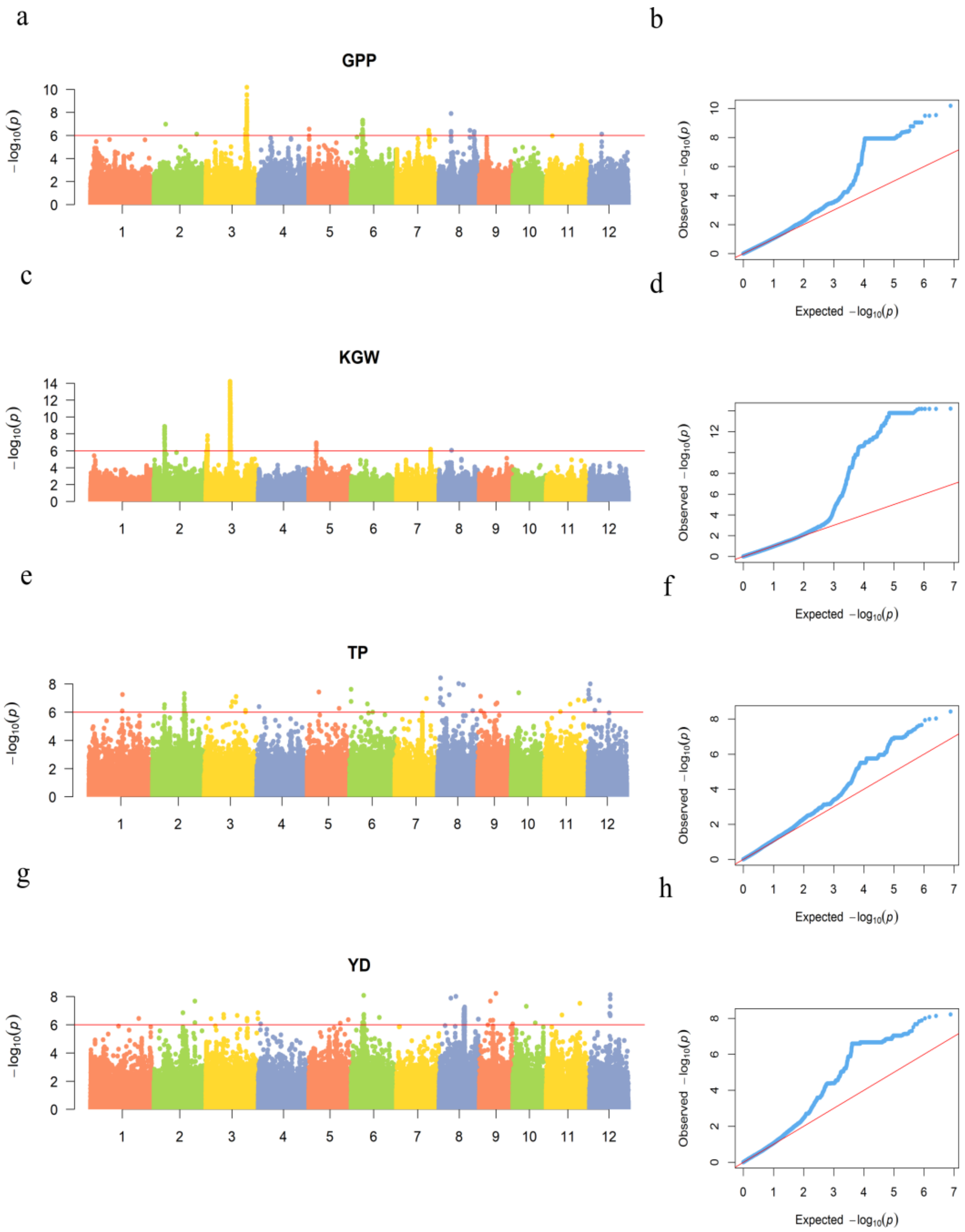

Figure S3. GWAS results of GPP, KGW, TP and YD in Sanya. (a, b) Manhattan plots and quantile-quantile plots of GPP. (c, d) Manhattan plots and quantile-quantile plots of KGW. (e, f) Manhattan plots and quantile-quantile plots of TP. (g, h) Manhattan plots and quantile-quantile plots of YD. The genome-wide significant *P* value threshold $P<10^{-6}$ is indicated by a horizontal line.

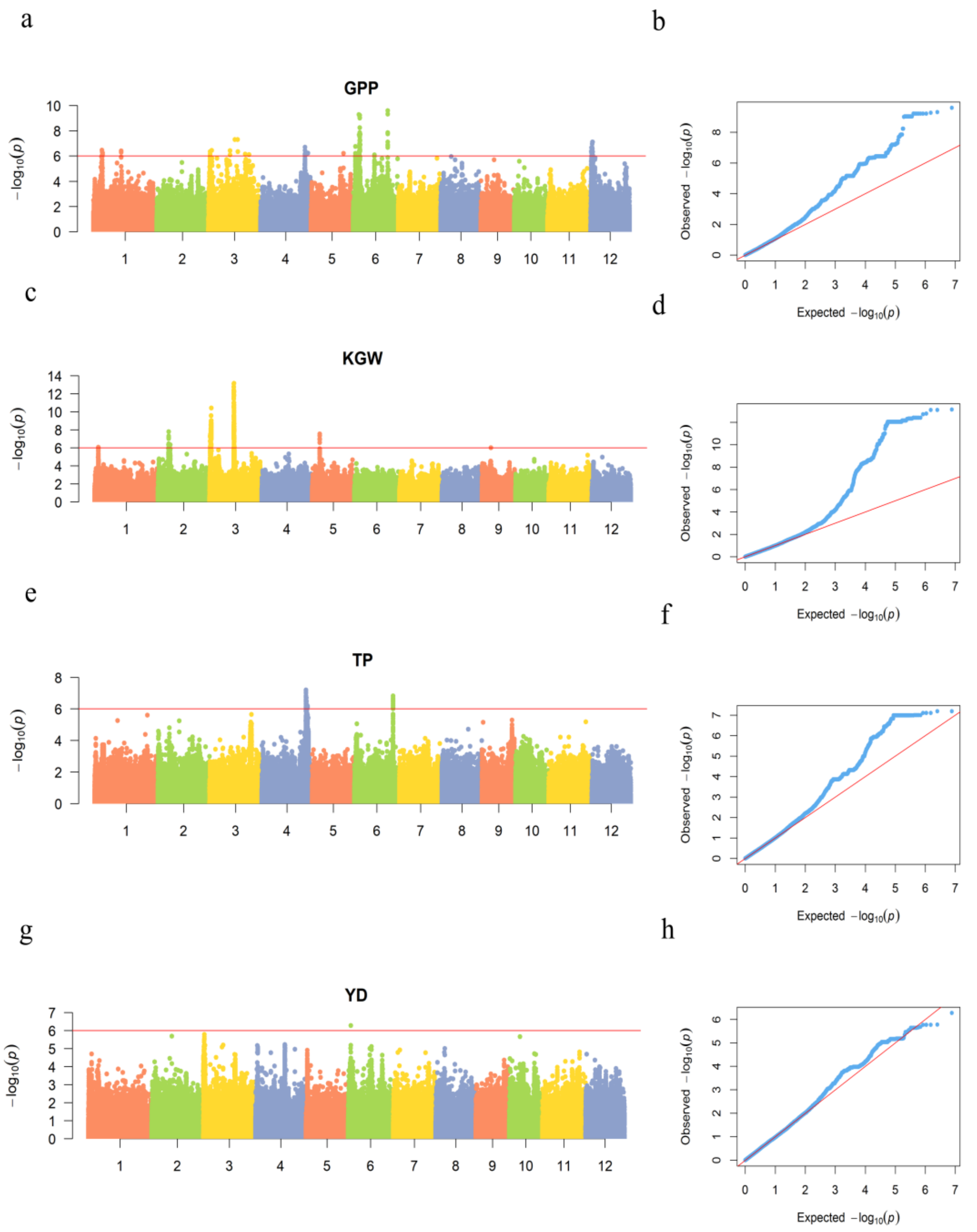

Figure S4. GWAS results of GPP, KGW, TP and YD in Hangzhou. (a, b) Manhattan plots and quantile-quantile plots of GPP. (c, d) Manhattan plots and quantile-quantile plots of KGW. (e, f) Manhattan plots and quantile-quantile plots of TP. (g, h) Manhattan plots and quantile-quantile plots of YD. The genome-wide significant *P* value threshold $P<10^{-6}$ is indicated by a horizontal line.

Table S2 Candidate genes detected by meta-GWAS

Trait	SNP	Chromosome	Position	Gene	Gene symbol
GPP	chr04_34254372	4	34254372	*Os04g0670900*	*sh4*
GPP	chr04_5790006	4	5790006	*Os04g0186400*	*OsPT4*
GPP	chr05_7226049	5	7226049	*Os05g0213500*	*OsPYL11*
GPP	chr07_23525330	7	23525330	*Os07g0580500*	*OsBZR1*
GPP	chr08_25258525	8	25258525	*Os08g0509600*	*OsSPL14*
GPP	chr12_22633431	12	22633431	*Os12g0555600*	*OsCD1*
KGW	chr01_5531015	1	5531015	*Os01g0201250*	*D-h*
KGW	chr02_7694050	2	7694050	*Os02g0234200*	*FUWA*
KGW	chr02_7960702	2	7960702	*Os02g0244100*	*GW2*
KGW	chr02_832725	2	832725	*Os02g0115900*	*OsBip1*
KGW	chr02_409112	2	409112	*Os02g0106966*	
KGW	chr03_33044576	3	33044576	*Os03g0793500*	*EL1*
KGW	chr07_23485525	7	23485525	*Os07g0580500*	*OsBZR1*
KGW	chr08_25422478	8	25422478	*Os08g0509600*	*OsSPL14*
TP	chr02_409112	2	409112	*Os02g0106966*	
TP	chr04_33048526	4	33048526	*Os04g0648800*	*HAF1*
TP	chr06_1578700	6	1578700	*Os06g0127800*	*D62*
TP	chr06_27043225	6	27043225	*Os06g0660200*	*OsPIN2*

Note: All the SNP markers are named after the chromosome _ position

Table S3 Superior alleles of the SNPs with affected yield

Effect	Trait	SNP	Allele	*P*-value	Superior allele
Direct	YD	chr01_9982003	G/A	2.08E-07	AA
	YD	chr06_1780896	T/C	9.74E-07	TC
	YD	chr06_1974504	A/T	6.60E-08	TT
Indirect	GPP	chr05_7226049	A/T	5.49E-03	TT
	KGW	chr03_33060865	T/C	2.72E-02	TC
	TP	chr02_21604477	T/C	4.31E-02	CC/TC
	TP	chr06_1578700	A/T	3.21E-04	TT
	TP	chr11_26492375	T/C	4.34E-02	CC

Table S4 The average yield performance (g) with different number of superior alleles

Effect	No. of superior alleles				
	0	1	2	3	4
Direct	41.29	44.26			
Indirect	42.22	42.75	42.76	44.54	47.49
Direct plus indirect	40.97	41.64	43.64	43.73	47.15
